# Supplementary material for: Implementing Rounding Checklists in a Pediatric Oncologic Intensive Care Unit
Source: Children (Basel). 2022 Apr 18;9(4):580. doi: 10.3390/children9040580 (PMC9025551; doi:10.3390/children9040580)
Supplement: Supplementary file 1 [file children-09-00580-s001.zip › children-1626371-supplementary.pdf]

children

MDPI

Article

Implementing Rounding Checklists in a Pediatric Oncologic Intensive Care Unit

Gardner MK[1], Amado PJ[1], Baig MU[2], Mohiuddin S[2], Harden A[2], Ewing LJ[3], Razvi S[3], Cortes JA[3], Mejia R[3], Petropoulos D[4], Tewari P[4] and Ahmad AH[3*].

[1] Pediatric Critical Care, Division of Nursing, The University of Texas MD Anderson Cancer Center, Houston, TX 77030, USA
[2] Pediatric Oncology Fellowship Program, Department of Pediatrics, The University of Texas MD Anderson Cancer Center, Houston, TX, 77030, USA
[3] Section of Pediatric Critical Care, Department of Pediatrics, The University of Texas MD Anderson Cancer Center, Houston, TX 77030, USA
[4] Section of Pediatric Stem Cell Transplantation and Cellular Therapy, Department of Pediatrics, The University of Texas MD Anderson Cancer Center, Houston, TX, 77030, USA
* Correspondence: ahahmad@mdanderson.org

Citation: Gardner, M.K.; Amado, P.J.; Baig, M.U.; Mohiuddin, S.; Harden, A.; Ewing, L.J.; Razvi, S.; Cortes, J.A.; Mejia, R.; Petropoulos, D.; Tewari, P.; Ahmad, A.H. Implementing Rounding Checklists in a Pediatric Oncologic Intensive Care Unit. *Children* **2022**, *9*, 580. https://doi.org/10.3390/children9040580

Academic Editor: Mara Leimanis Laurens

Received: 20 February 2022
Accepted: 12 April 2022
Published: 18 April 2022

Publisher's Note: MDPI stays neutral with regard to jurisdictional claims in published maps and institutional affiliations.

Copyright: © 2022 by the authors. Licensee MDPI, Basel, Switzerland. This article is an open access article distributed under the terms and conditions of the Creative Commons Attribution (CC BY) license (https://creativecommons.org/licenses/by/4.0/).

Supplemental figures: Central line associated blood stream infection (CLABSI), catheter associated urinary tract infection (CAUTI), and ventilator associated pneumonia (VAP) data

Supplemental Figures S1–S3 CLABSI, CAUTI and VAP results. There were 1222 central line days, 312 urinary catheter days, and 123 ventilator days in our pediatric oncologic ICU during this time period. We sustained a rate of zero for CLABSI, CAUTI and VAP during the 12-month period prior to, during and post-intervention (October 2019–September 2020).

Supplemental Figure S1. CLABSI Data.

	Central Line Days	Hospital-Acquired CLABSI	CLABSI per 1000 Central Line Days
Nursing Cohort: Pediatric (cont'd)	5,590	8	1.43
Nursing G9NW Pedi Intensive	1,222	0	0.00

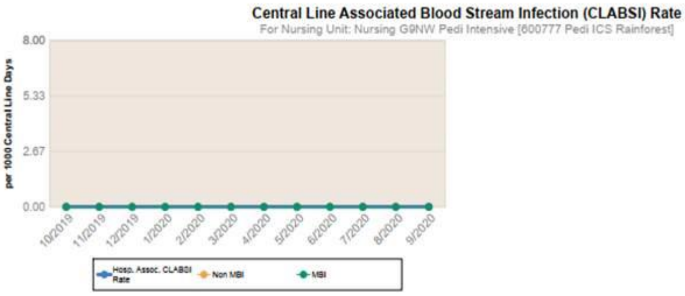

	Oct 2019	Nov 2019	Dec 2019	Jan 2020	Feb 2020	Mar 2020	Apr 2020	May 2020	Jun 2020	Jul 2020	Aug 2020	Sep 2020
Central Line Days	163	79	97	83	104	120	116	112	75	87	97	89
CLABSI Rate	0.00	0.00	0.00	0.00	0.00	0.00	0.00	0.00	0.00	0.00	0.00	0.00
CLABSI Cases	0	0	0	0	0	0	0	0	0	0	0	0
MBI Rate	0.00	0.00	0.00	0.00	0.00	0.00	0.00	0.00	0.00	0.00	0.00	0.00
MBI Cases	0	0	0	0	0	0	0	0	0	0	0	0
non MBI Rate	0.00	0.00	0.00	0.00	0.00	0.00	0.00	0.00	0.00	0.00	0.00	0.00
non MBI Cases	0	0	0	0	0	0	0	0	0	0	0	0

Supplemental Figure S2. CAUTI Data.

Hospital-Acquired Catheter-Associated Urinary Tract Infections - 10/1/2019 to 9/30/2020	Urinary Catheter Days	Hospital-Acquired CAUTI	Hosp-Acquired CAUTI per 1000 Urinary Catheter Days
**Nursing Cohort: Pediatric** (cont'd)	**541**	**0**	**0.00**
**Nursing G9NW Pedi Intensive**	**312**	**0**	**0.00**

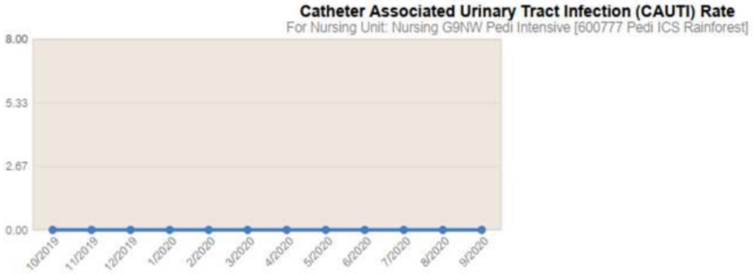

	Oct 2019	Nov 2019	Dec 2019	Jan 2020	Feb 2020	Mar 2020	Apr 2020	May 2020	Jun 2020	Jul 2020	Aug 2020	Sep 2020
**Rate**	0.00	0.00	0.00	0.00	0.00	0.00	0.00	0.00	0.00	0.00	0.00	0.00
**Urinary Catheter Days**	37	11	42	22	35	46	16	32	4	15	28	24
**Hospital-Acquired CAUTI**	0	0	0	0	0	0	0	0	0	0	0	0

Supplemental Figure S3. VAP Data.

Pediatric Hospital-Acquired Ventilator Associated Pneumonia - 10/1/2019 to 9/30/2020	Vent Days	Pediatric Hospital-Acquired VAP	Pediatric Hosp-Acquired VAP per 1000 Vent Days
Nursing Cohort: Pediatric	123	0	0.00

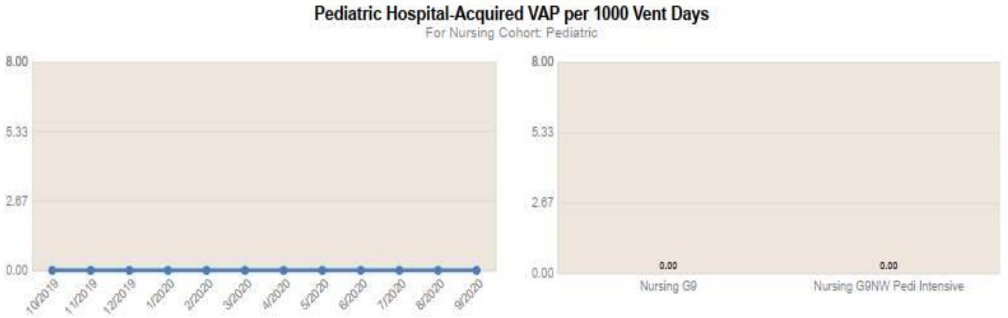

For Nursing Cohort: Pediatric

	Oct 2019	Nov 2019	Dec 2019	Jan 2020	Feb 2020	Mar 2020	Apr 2020	May 2020	Jun 2020	Jul 2020	Aug 2020	Sep 2020
**Rate**	0.00	0.00	0.00	0.00	0.00	0.00	0.00	0.00	0.00	0.00	0.00	0.00
**Vent Days**	7	1	1	11	7	9	30	37	1	0	10	9
**Pediatric Hospital-Acquired VAP**	0	0	0	0	0	0	0	0	0	0	0	0
